# Case report: A cystic capillary hemangioma located at the conus medullaris mimicking hemangioblastoma

**DOI:** 10.3389/fneur.2024.1350780

**Published:** 2024-03-28

**Authors:** Jiachen Sun, Jiuhong Li, Ziba Ayi, Feilong Yang, Junlin Hu, Xuhui Hui, Haifeng Chen, Jiaojiang He

**Affiliations:** ^1^Department of Neurosurgery/Department of Cardiovascular Surgery, West China Hospital of Sichuan University, Chengdu, China; ^2^West China School of Medicine, Sichuan University, Chengdu, China; ^3^Department of Neurosurgery, People’s Hospital of Renshou, Meishan, China

**Keywords:** capillary hemangioma, cystic, spinal, conus medullaris, mimicking, hemangioblastoma

## Abstract

Capillary hemangiomas, usually found in skin and mucosal tissues, are rarely encountered within the spinal cord, presenting a significant diagnostic challenge. We report a rare case of intradural extramedullary capillary hemangioma at the conus medullaris in a 66-year-old female patient. Our initial diagnosis leaned towards a cystic hemangioblastoma based on MRI findings due to the presence of cystic formation with an enhanced mural nodule. However, surgical exploration and subsequent pathological examination revealed the lesion as a capillary hemangioma. To the authors’ knowledge, this case may represent the first documented instance of a spinal capillary hemangioma that mimics a cystic hemangioblastoma.

## 1 Introduction

Capillary hemangiomas, benign vascular tumors typically found in skin and mucosal tissues, are exceedingly rare in the spinal cord, with around 100 cases documented so far. These includes about 30 epidural (1), 60 intradural extramedullary (2), and fewer than 20 intramedullary cases (3), posing a significant diagnostic challenge. On magnetic resonance imaging (MRI), spinal capillary hemangioma (SCH) typically appears as round, well-demarcated solid lesion that is isointense on T1-weighted images and hyperintense on T2-weighted images (4). Pathologically, SCH exhibits a lobular architecture of densely packed capillary-sized vessels separated by fibrous tissue septa (5), and is histologically indistinguishable from the cutaneous capillary hemangioma (6). In this report, we present a unique case of an intradural extramedullary capillary hemangioma at the conus medullaris, characterized by an enhanced mural nodule with cystic formation on MRI, closely resembling the imaging features of cystic hemangioblastomas. To our knowledge, such a presentation has not been previously described in English medical literature.

## 2 Case description

**Figure 1:**
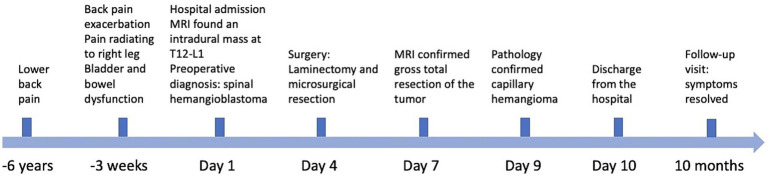
Time course and clinical findings of the patient.

A 66-year-old Asian female presented with six-year history of lower back pain (Figure 1), which had exacerbated over the past three weeks, radiating to her right buttock and thigh. She also reported a recent onset of bladder and bowel dysfunction. Physical examination noted hyperesthesia in the lower back, right buttock and posterior thigh, with hypoesthesia on the sole of her left foot. Muscle strength and reflexes were found to be normal.

**Figure 2:**
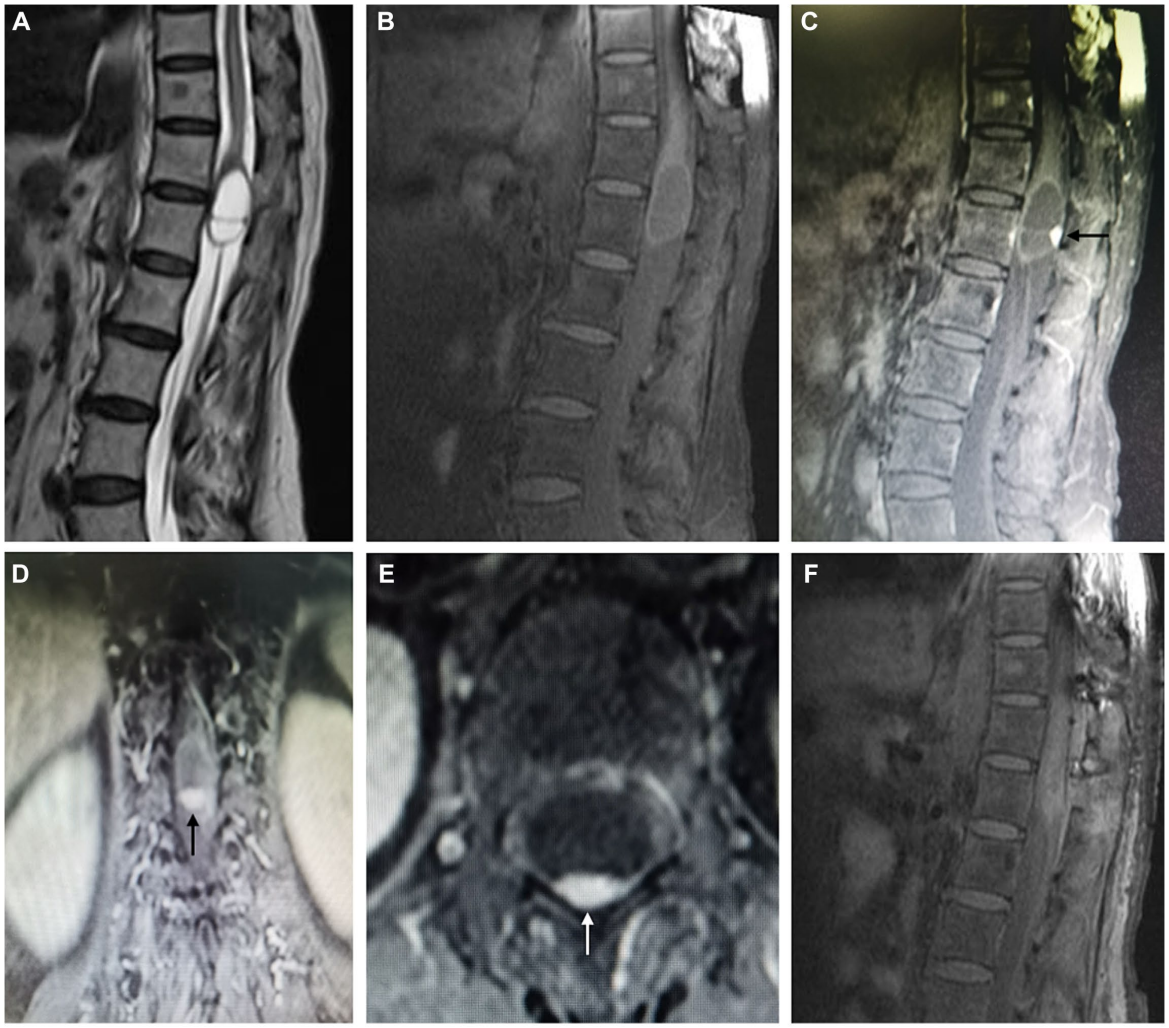
Magnetic resonance imaging. Preoperative MR T2 and T1-weighted images demonstrate an intradural septate cystic mass with a similar intensity of cerebrospinal fluid located at the terminal end of the conus medullaris, with a small posterior nodular lesion **(A,B)**. Preoperative MR T1-weighted contrast-enhanced images demonstrate homogenous enhancement of posterior nodule (arrow) and cystic wall **(C–E)**. Postoperative MR T1-weighted contrast-enhanced image demonstrates the gross total resection of the mass **(F)**.

Lumbar MRI revealed an intradural septate cystic mass located at the T12-L1 level (Figures 2A,B). The cyst was separated by a thin septum and presented a similar intensity of cerebrospinal fluid (CSF). Additionally, a small nodular lesion was located on the posterior wall of the cyst, which was isointense on both T2 and T1-weighted images. After gadolinium administration, the nodule exhibited strong homogenous enhancement and the cystic wall was also homogeneously enhanced (Figures 2C–E). No signs of syringomyelia or adjacent spinal cord edema were observed. These imaging features led to an initial diagnostic consideration of a cystic hemangioblastoma.

**Figure 3:**
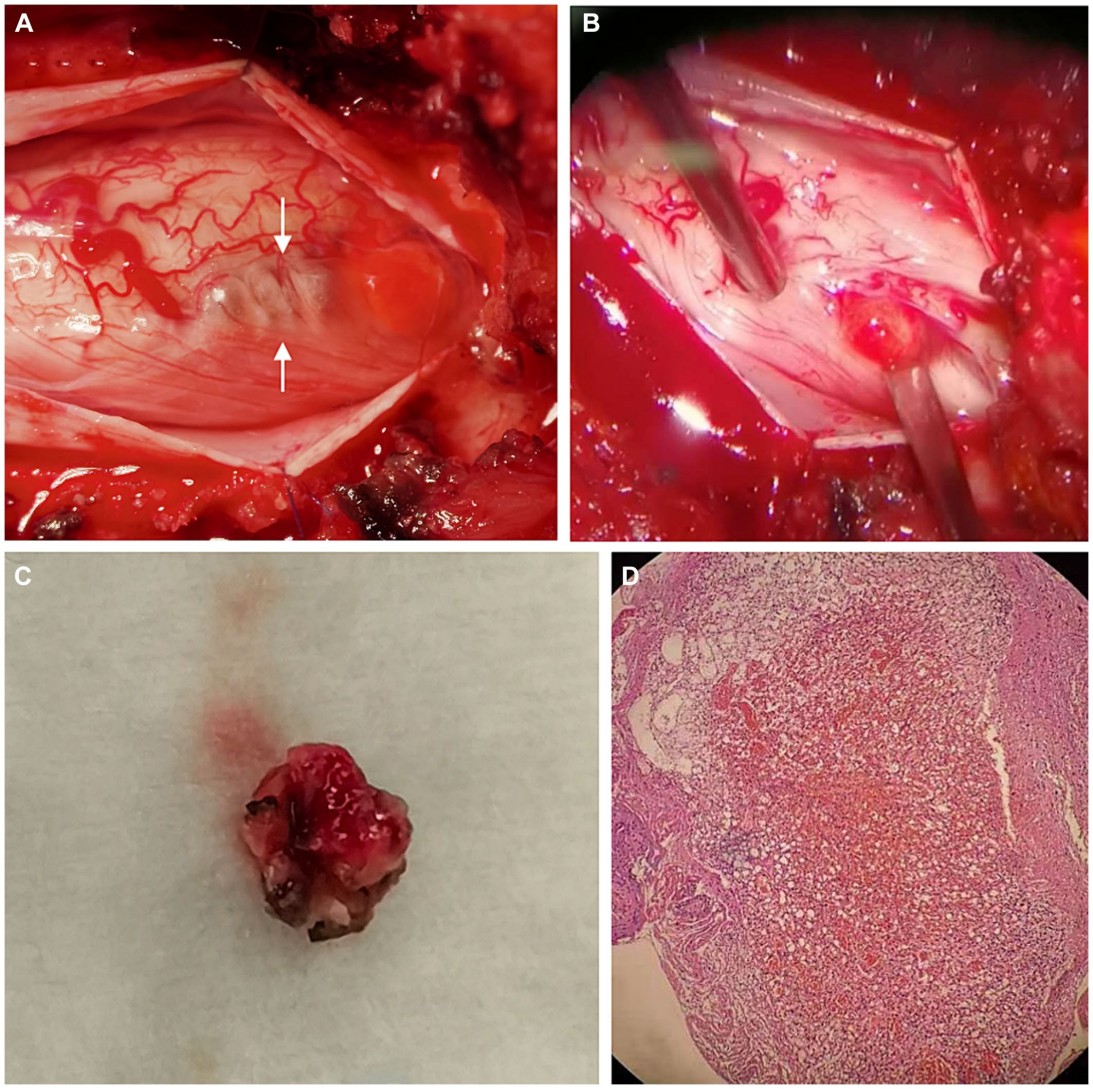
Operative and pathological findings. During operation, a soft blister-like mass with cystic formation (arrows) was detected **(A)**. We found that the lesion arose from the cauda equina **(B)**. The mural nodule of the tumor was resected en bloc **(C)**. Hematoxylin & eosin (magnification, ×20) **(D)** showed lobular arrangement of numerous, tightly packed, capillary-sized vessels, indicating a capillary hemangioma.

The patient underwent posterior T12-L1 laminectomy. The dura was opened paramedially, exposing an intradural soft blister-like mass with cyst formation at the conus medullaris (Figures 3A,B). The tumor had an abundant blood supply and was tightly adhesive to the surrounding tissues. The cyst was drained, and the feeding vessels were coagulated and cut, followed by en-bloc resection of the tumor (Figure 3C).

Pathological examination showed a lobular arrangement of numerous, tightly packed, capillary-sized vessels, confirming the diagnosis of a capillary hemangioma (Figure 3D). The patient’s postoperative course was uneventful, with a repeated contrast-enhanced MRI conducted three days postoperatively confirmed the gross total resection of the tumor (Figure 2F). At a follow-up visit ten months later, the patient reported complete resolution of previous symptoms and no new complaints, demonstrating the effectiveness of the surgical intervention.

## 3 Discussion

SCHs are slow-growing tumors more commonly observed in middle-aged individuals, with a slightly higher prevalence in males (2, 3). However, epidural SCHs exhibit a slight female predominance (1). Patients with SCHs usually present with chronic, progressive back pain and radicular pain due to compression of the spinal cord and nerves, accompanied by sensory and motor deficits, as well as bowel and bladder dysfunction. In our case, the clinical manifestations of the cystic SCH were similar to those seen in solid SCHs. We hypothesize that the cyst expansion played a crucial role in the progression of symptoms.

In addition to the typical presentations, previous cases reported atypical clinical manifestations of SCHs. Notably, there have been reports of acute onset of symptoms following trauma (2, 7, 8). Moreover, epidural SCHs may extend intrathoracically through neural foramina, potentially being misidentified as pulmonary neoplasia (9–12). A particularly unique case involved a 42-year-old man experiencing back pain and symptoms of increased intracranial pressure, including headache, pulsatile tinnitus, and visual disturbances. These were ultimately attributed to a capillary hemangioma in the cauda equina (13). These cases underscore the unexpected clinical spectrum associated with SCHs.

Diagnosing SCHs poses significant challenges due to their rarity. Although MRI plays a crucial role in diagnosis, its findings can be inconclusive, leading to potential misdiagnoses. This ambiguity arises because the imaging characteristic of SCHs may closely mimic those of other spinal tumors, such as schwannomas or meningiomas (9).

SCHs are typically well-defined lesions, predominantly located in the posterior portion of the spinal canal. On MRI, they demonstrate isointensity on T1-weighted images and hyperintensity on T2-weighted images relative to the spinal cord, with avid homogenous enhancement after contrast administration (14). Larger tumors may exhibit vascular flow voids, indicative of their abundant blood supply (15). Associated syringomyelia has been observed in intramedullary cases (3). Although rare, edema has also been reported (16).

Hemangioblastoma is a highly vascularized benign tumor of the CNS that can be found in the cerebellum, spinal cord, brainstem, and supratentorial area (17). Although it is the most common primary neoplasm in the adult cerebellum, hemangioblastoma represents a rare entity within the spinal cord, accounting for 1–5% of all spinal tumors (18, 19). Spinal hemangioblastomas (SHBs) are typically located on the dorsal surface of the spinal cord with the majority being intramedullary; only about 20% are primarily extramedullary (20). These tumors typically manifest as a large intramedullary cyst with a homogeneously contrast-enhanced mural nodule. On MRI, SHB is usually hypointense to isointense on T1-weighted images and isointense to hyperintense on T2-weighted images (21). Smaller tumors are often isointense and difficult to differentiate from the spinal cord, whereas larger tumors can exhibit flow voids resulting from prominent vessels and demonstrate heterogenous contrast enhancement. Surrounding edema within the cord can be seen, and syringomyelia or cyst formation were observed in half of the cases. Compared to CSF, the cystic fluid in these tumors may appear slightly hyperintense on T1-weighted images and more hyperintense on T2-weighted images due to its higher protein content (22).

SCH with cystic formation is extremely rare, with only a single case documented previously. Holtzman et al. (23) reported a case involving a 55-year-old female who presented with low-back pain, sciatica, and paresthesias over a year. The capillary hemangioma was located at L4, and a loculated cyst formation was found caudally to the tumor during the surgery. In our case, the cyst constituted a significant portion of the mass volume and featured an enhanced mural nodule, closely resembling the typical radiological characteristics of a cystic SHB (24). To the best of our knowledge, this presentation has not been previously described in English medical literature.

Surgical intervention is generally regarded as the preferred treatment strategy for SCHs, with most patients achieving favorable outcomes following gross total resection of the tumor (1–3). However, instances of tumor regrowth have been documented, highlighting the necessity for vigilant postoperative monitoring. Kaneko et al. (25) described a case of thoracic SCH that exhibited rapid regrowth six months after gross total resection. Wu et al. (3) reported on the regrowth of a small remnant of an intramedullary SCH ten months after a subtotal resection. These cases underscore the potential need for considering adjuvant therapies in the management of SCHs. A recent case has demonstrated the effectiveness of multimodal therapies; following an initial subtotal resection due to the tumor’s strong adherence to the spinal cord, stereotactic cyber knife treatment was administered one month after surgery. Subsequent MRI scans confirmed a reduction of the residual tumor size with evidence of recurrence after two years (26).

## 4 Conclusion

We present a novel case of a cystic spinal capillary hemangioma, which closely mimics a cystic hemangioblastoma on MRI. This case report contributes the current knowledge on the presentations of spinal capillary hemangiomas. It underscores the importance of considering capillary hemangioma in the differential diagnosis of a cystic spinal cord lesion with an enhancing mural nodule, despite its rarity. This case highlights the diagnostic challenges posed by such a rare entity, especially when MRI findings may be inconclusive, emphasizing the need for meticulous interpretation.

## Data availability statement

The datasets presented in this article are not readily available because of ethical and privacy restrictions. Requests to access the datasets should be directed to the corresponding authors.

## Ethics statement

The studies involving humans were approved by the Institutional Ethics Committee for Clinical Research of West China Hospital, Sichuan University. The studies were conducted in accordance with the local legislation and institutional requirements. Written informed consent for participation was not required from the participants or the participants' legal guardians/next of kin in accordance with the national legislation and institutional requirements. Written informed consent was obtained from the individual(s) for the publication of any potentially identifiable images or data included in this article.

## Author contributions

JS: Conceptualization, Data curation, Investigation, Writing – original draft. JL: Conceptualization, Data curation, Investigation, Writing – original draft. ZA: Writing – original draft. FY: Writing – review & editing. JuH: Resources, Writing – review & editing. XH: Writing – review & editing. HC: Funding acquisition, Supervision, Writing – review & editing. JiH: Funding acquisition, Supervision, Writing – review & editing.
